# Geography of Africa biomedical publications: An analysis of 1996–2005 PubMed papers

**DOI:** 10.1186/1476-072X-6-46

**Published:** 2007-10-10

**Authors:** Olalekan A Uthman, Mubashir B Uthman

**Affiliations:** 1 Center for Evidence-Based Global Health, Save the Youth Initiative, Nigeria; 2 Department of Community Health and Epidemiology, University of Ilorin, Nigeria

## Abstract

**Background:**

Scientific publications play an important role in scientific process providing a key linkage between knowledge production and use. Scientific publishing activity worldwide over the past decades shows that most countries in Africa have low levels of publication. We sought to examine trends and contribution of different Africa subregions and individual countries as represented by the articles indexed by PubMed between 1996 and 2005.

**Results:**

Research production in Africa is highly skewed; South Africa, Egypt, and Nigeria make up a striking 60% of the total number of articles indexed by PubMed between 1996 and 2005. When adjusted for population size smaller countries, such as The Gambia, Gabon and Botswana, were more productive than Nigeria and Kenya. The Gambia and Eritrea had better records when total production was adjusted for gross domestic product. The contribution of Africa to global research production was persistently low through the period studied.

**Conclusion:**

In this study, we found that most populous and rich countries (such as South Africa, Egypt, and Nigeria) have correspondingly higher research production; but smaller countries can be productive. We noted continuous increases and reassuring trends in the production of research articles from all African subregions during the period 1996 – 2005. However, contribution of Africa to global research production was limited.

## Background

Scientific publications play an important role in the scientific process providing a key linkage between knowledge production and use. Perceptions of research productivity have been reported to vary widely [1-4]. In general, the USA and Europe are the leaders of global biomedical research [5-8]. Scientific publishing activity worldwide over the past decades shows that most countries in Africa have low levels of publication [9].

There are number of useful bibliometric studies available which have focused in part with Africa or with specific African countries [10-21]. However, as noted by Tijssen [21] some lacks comprehensive cross-country comparison and their scope and coverage of the research literature is too constrained to serve as a broad cross-country analyses at African level. The most recent is a bibliometric analysis of research output of African-based authors using Thompson scientific citation index [21]. Tijssen [21] found that Africa's contribution to global knowledge production has slipped slightly during 1990s. Tijssen [21] state in his comprehensive discussion of this issue that the main reason for this decline is the lack of the resources in many African countries, and willingness to invest in infrastructure and modern equipment to retain workers at Universities, research laboratories and health institutions [22].

According to a survey of 158 African medical journal editors from 33 countries conducted by Siegfried et al [23], most African medical journals are owned by academic institutions, have a small circulations, lack adequate funding, have a difficulty maintaining publication frequency, and are rarely indexed in major databases. Most of the journals have minimal staff to coordinate both the review and production processes [23,24]. Access to technological tools and other equipment and supplies to ease the work is not always possible [25]. Another difficulty facing African journals is dissemination of their content to other parts of the world [25]. Most information published in African journals never leaves its home borders because these journals are largely not included in major bibliographic databases like the National Library of Medicine's (NLM) MEDLINE [25,26]. Only 31 of the 4,900 journals indexed by MEDLINE are from Africa [25]. The difficulties in research, publication, and information access facing the least-developed are profound and seem almost intractable [26]. In this bibliometric analysis, we sought to examine trend and contribution of different African subregions and individual countries as it is represented by the articles indexed by PubMed between 1996 and 2005, as well as the relationship of research production of individual countries with their population and gross domestic product.

## Methods

### Search strategy

We searched PubMed database in May 2007 to obtain the research volume of each country from January 1, 1996 to December 31, 2005. Articles originating from each country and published between 1996 and 2005 were generated by selecting the advanced-search option and then selecting the "publication date" field. Next, the "affiliation" field was searched for each country. The names of the countries were imputed in their different possible forms: Côte d'Ivoire and Ivory Coast for Côte d'Ivoire, and both Swasiland and Swaziland for Swaziland, for example. Some names of countries are also names of parts of other countries: Benin and Niger, for example are name of a place in Nigeria. To avoid errors arising from this, appropriate commands were used [i.e. (Niger [AD] NOT Nigeria)].

The number of biomedical articles originating from each of the countries over the period 1996–2005 was used as an index of total biomedical research production. 'Global research production' is represented in this paper by the total number of articles indexed in PubMed per year from 1996 to 2005. To allow for weighted comparison among the countries of origin we calculated the ratio of the number of publications from a certain country to the number of inhabitants in that country. We also calculated the ratio between number of publications and gross domestic product (GDP) in billions of US dollars for 2005. The resident population and GDP of the countries were retrieved from the United Nations World Prospect 2006 revision [27] and annual statistic reviews of the World Bank database [28] respectively. From these data, we composed three top-20 lists: one for 20 countries with the highest absolute number of publications over the study period; second list for countries with the highest number of publications relative to their number of inhabitants; and the third list for countries with the highest number of publications per GDP.

### Africa subregions

For this study, Africa was divided into five subregions based on combination of geographic, economic, and scientific criteria [29]. The five subregions are Northern Africa, Western Africa, Middle Africa, Eastern Africa, and Southern Africa.

### Statistical analyses

Time trends in the research production over the period from 1996 to 2005 were analyzed by Poisson regression models using the absolute research product as outcome variable. This method allows for estimation of trends across individual calendar years to obtain average annual percentage changes. The Poisson regression procedure fits a model of the following form:

ln(p) = a + β*(t)

where p equals number of articles per year, log is the natural log, a is the intercept, β is the trend, and t is the year – year is given as 0, 1, 2, . 9 (year 0 is 1996, year 1 is 1997, and so on to 2005). Average annual percentage changes (AAPC) were calculated using the following formula:

AAPC = 100 * (exp (β) - 1)

We used the average annual percentage change of scientific output of different countries to calculate future performance using a projection model. To examine the change in trends by years, three periods were considered 1996 to 2000, 2001 to 2005, and 1996 to 2005. Percent relative growth was calculated for each country as follows:

percent relative growth=(number of publications in 2005number of publications in 2005−number of publications in 1996)×100
 MathType@MTEF@5@5@+=feaafiart1ev1aaatCvAUfKttLearuWrP9MDH5MBPbIqV92AaeXatLxBI9gBaebbnrfifHhDYfgasaacH8akY=wiFfYdH8Gipec8Eeeu0xXdbba9frFj0=OqFfea0dXdd9vqai=hGuQ8kuc9pgc9s8qqaq=dirpe0xb9q8qiLsFr0=vr0=vr0dc8meaabaqaciaacaGaaeqabaqabeGadaaakeaacqWGWbaCcqWGLbqzcqWGYbGCcqWGJbWycqWGLbqzcqWGUbGBcqWG0baDcqqGGaaicqWGYbGCcqWGLbqzcqWGSbaBcqWGHbqycqWG0baDcqWGPbqAcqWG2bGDcqWGLbqzcqqGGaaicqWGNbWzcqWGYbGCcqWGVbWBcqWG3bWDcqWG0baDcqWGObaAcqGH9aqpcqGGOaakdaWcaaqaaiabd6gaUjabdwha1jabd2gaTjabdkgaIjabdwgaLjabdkhaYjabbccaGiabd+gaVjabdAgaMjabbccaGiabdchaWjabdwha1jabdkgaIjabdYgaSjabdMgaPjabdogaJjabdggaHjabdsha0jabdMgaPjabd+gaVjabd6gaUjabdohaZjabbccaGiabdMgaPjabd6gaUjabbccaGiabikdaYiabicdaWiabicdaWiabiwda1aqaaiabd6gaUjabdwha1jabd2gaTjabdkgaIjabdwgaLjabdkhaYjabbccaGiabd+gaVjabdAgaMjabbccaGiabdchaWjabdwha1jabdkgaIjabdYgaSjabdMgaPjabdogaJjabdggaHjabdsha0jabdMgaPjabd+gaVjabd6gaUjabdohaZjabbccaGiabdMgaPjabd6gaUjabbccaGiabikdaYiabicdaWiabicdaWiabiwda1iabgkHiTiabd6gaUjabdwha1jabd2gaTjabdkgaIjabdwgaLjabdkhaYjabbccaGiabd+gaVjabdAgaMjabbccaGiabdchaWjabdwha1jabdkgaIjabdYgaSjabdMgaPjabdogaJjabdggaHjabdsha0jabdMgaPjabd+gaVjabd6gaUjabdohaZjabbccaGiabdMgaPjabd6gaUjabbccaGiabigdaXiabiMda5iabiMda5iabiAda2aaacqGGPaqkcqGHxdaTcqaIXaqmcqaIWaamcqaIWaamaaa@C387@

In addition, percent share of world research output was calculated for each year as follows:

percent share of world research output (per year)=(number of articles from all Africa subregionstotal number of articles index in PubMed)×100
 MathType@MTEF@5@5@+=feaafiart1ev1aaatCvAUfKttLearuWrP9MDH5MBPbIqV92AaeXatLxBI9gBaebbnrfifHhDYfgasaacH8akY=wiFfYdH8Gipec8Eeeu0xXdbba9frFj0=OqFfea0dXdd9vqai=hGuQ8kuc9pgc9s8qqaq=dirpe0xb9q8qiLsFr0=vr0=vr0dc8meaabaqaciaacaGaaeqabaqabeGadaaakeaacqWGWbaCcqWGLbqzcqWGYbGCcqWGJbWycqWGLbqzcqWGUbGBcqWG0baDcqqGGaaicqWGZbWCcqWGObaAcqWGHbqycqWGYbGCcqWGLbqzcqqGGaaicqWGVbWBcqWGMbGzcqqGGaaicqWG3bWDcqWGVbWBcqWGYbGCcqWGSbaBcqWGKbazcqqGGaaicqWGYbGCcqWGLbqzcqWGZbWCcqWGLbqzcqWGHbqycqWGYbGCcqWGJbWycqWGObaAcqqGGaaicqWGVbWBcqWG1bqDcqWG0baDcqWGWbaCcqWG1bqDcqWG0baDcqqGGaaicqGGOaakcqWGWbaCcqWGLbqzcqWGYbGCcqqGGaaicqWG5bqEcqWGLbqzcqWGHbqycqWGYbGCcqGGPaqkcqGH9aqpcqGGOaakdaWcaaqaaiabd6gaUjabdwha1jabd2gaTjabdkgaIjabdwgaLjabdkhaYjabbccaGiabd+gaVjabdAgaMjabbccaGiabdggaHjabdkhaYjabdsha0jabdMgaPjabdogaJjabdYgaSjabdwgaLjabdohaZjabbccaGiabdAgaMjabdkhaYjabd+gaVjabd2gaTjabbccaGiabdggaHjabdYgaSjabdYgaSjabbccaGiabdgeabjabdAgaMjabdkhaYjabdMgaPjabdogaJjabdggaHjabbccaGiabdohaZjabdwha1jabdkgaIjabdkhaYjabdwgaLjabdEgaNjabdMgaPjabd+gaVjabd6gaUjabdohaZbqaaiabdsha0jabd+gaVjabdsha0jabdggaHjabdYgaSjabbccaGiabd6gaUjabdwha1jabd2gaTjabdkgaIjabdwgaLjabdkhaYjabbccaGiabd+gaVjabdAgaMjabbccaGiabdggaHjabdkhaYjabdsha0jabdMgaPjabdogaJjabdYgaSjabdwgaLjabdohaZjabbccaGiabdMgaPjabd6gaUjabdsgaKjabdwgaLjabdIha4jabbccaGiabdMgaPjabd6gaUjabbccaGiabdcfaqjabdwha1jabdkgaIjabd2eanjabdwgaLjabdsgaKbaacqGGPaqkcqGHxdaTcqaIXaqmcqaIWaamcqaIWaamaaa@DF91@

Finally, the Pearson correlation analysis method was used to examine the association of 1) the research production with population 2) research production and GDP, and 3) absolute numbers of published articles between the different Africa subregions during the years of study period (1996 – 2005). For correlation analysis, number of publications, population size, and GDP were log transformed to linearise these associations. Data were processed and analyzed with Stata 9.0 [30] software.

## Results

### Cross-country analyses

A total of 37 467 articles indexed by PubMed within the period of 1996 to 2005 were described in this study. Table 1 shows the top 20 top-ranking countries in terms of relative contribution of each country to the total number of articles that were indexed by PubMed. First authors from South Africa, Egypt, and Nigeria produced highest number of articles. We also observed a continuous increase in the number of articles produced from most of the countries. The number of articles produced by first authors from Zimbabwe reduced from 123 in 1996 to 58 in 2005. Over the study period, the absolute number of publications from The Gambia hardly increased and the relative contribution dropped from 1.3% in 1996 to 0.6% by 2005. While the number of publications from South Africa increased from 2442 to 4699, its relative share of Africa biomedical publication declined from 34.0% in 1996 to 28.4% in 2005. Table 1 makes it abundantly clear that Nigeria has been able to generate noticeable relative growth (2094.7%) during the years 1996 – 2005.

**Table 1:** Number of articles indexed by PubMed from top 20 countries, percentage of Africa publication, relative growth, and Poisson regression analysis of annual percent change: 1996 – 2005

	***Number of articles (% within a calendar year)***			***Relative growth***	***Poisson regression trend analysis***		
	**1996 – 2005**	**1996**	**2005**	**(%)**	**β (SE)**	**AAPC**	**P – value**

South Africa	11218 (29.9)	829 (34.0)	1536 (28.4)	85.3	0.06 (0.003)	6.3	< 0.001
Egypt	6557 (17.5)	414 (17.0)	956 (17.7)	130.9	0.01 (0.004)	10.4	< 0.001
Nigeria	4795 (12.8)	38 (1.6)	834 (15.4)	2094.7	0.15 (0.005)	16.3	< 0.001
Kenya	1887 (5.0)	189 (7.7)	246 (4.6)	30.2	0.05 (0.008)	5.5	< 0.001
Tunisia	1027 (2.7)	27 (1.1)	267 (4.9)	888.9	0.27 (0.013)	31.7	< 0.001
Zimbabwe	945 (2.5)	123 (5.0)	58 (1.1)	-52.8	-0.09 (0.012)	-8.5	< 0.001
Senegal	875 (2.3)	65 (2.7)	117 (2.2)	80.0	0.04 (0.012)	3.6	0.003
Morocco	863 (2.3)	33 (1.4)	141 (2.6)	327.3	0.14 (0.013)	15.5	< 0.001
Ethiopia	860 (2.3)	79 (3.2)	116 (2.1)	46.8	0.08 (0.013)	8.1	< 0.001
Tanzania	761 (2.0)	71 (2.9)	107 (2.0)	188.4	0.06 (0.013)	6.4	<0.001
Uganda	761 (2.0)	43 (1.8)	124 (2.3)	50.7	0.11 (0.013)	11.6	<0.001
Ghana	691 (1.8)	50 (2.1)	94 (1.7)	88.0	0.06 (0.013)	5.9	<0.001
Ivory coast	516 (1.4)	24 (1.0)	55 (1.0)	129.2	0.04 (0.015)	4.7	0.003
Cameroon	512 (1.4)	24 (1.0)	83 (1.5)	245.8	0.14 (0.016)	15.2	<0.001
Malawi	450 (1.2)	34 (1.4)	57 (1.1)	67.6	0.09 (0.017)	9.2	<0.001
Sudan	442 (1.2)	34 (1.4)	68 (1.3)	100.0	0.08 (0.017)	9.9	<0.001
Guinea	428 (1.1)	65 (2.7)	33 (0.6)	-49.2	-0.03 (0.017)	-3.3	0.044
Burkina Faso	365 (1.0)	22 (0.9)	39 (0.7)	77.3	0.05 (0.018)	4.9	0.009
Eritrea	337 (0.9)	34 (1.4)	54 (1.0)	58.8	0.15 (0.020)	16.6	<0.001
The Gambia	335 (0.9)	31 (1.3)	33 (0.6)	6.5	0.01 (0.019)	0.8	0.669

β indicates regression coefficient (trend); SE standard error of β, AAPC average annual percentage change

Poisson regression analysis confirmed continuous increase in the production of research articles from most of the Africa countries during the period 1996 – 2005 (Table 2). Tunisia had the most significant average annual percent change AAPC (31.7%, p <.001). We observed decline in research production in Zimbabwe (AAPC -8.5%, p <.001) and Guinea (AAPC -3.3%, p = .044). Using a projection model, we estimated that Tunisia, Eritrea and Nigeria would reach South Africa's production level in 8, 43 and 5 years respectively holding trends constant.

**Table 2:** Number of articles indexed by PubMed from different Africa subregions for the period of 1996 to 2005

*Number of articles ****[% percentage within calendar year] ****(% share of world research output)*											
**Subregions**	**1996 – 2005**	**1996**	**1997**	**1998**	**1999**	**2000**	**2001**	**2002**	**2003**	**2004**	**2005**

Eastern Africa	6810	662	581	486	588	631	719	742	772	782	847
	**[18.2] **(0.13)	**[27.1] **(0.15)	**[20.5] **(0.13)	**[16.6] **(0.11)	**[18.7] **(0.12)	**[17.9] **(0.12)	**[18.1] **(0.13)	**[18.0] **(0.13)	**[17.4] **(0.13)	**[16.6] **(0.12)	**[15.6] **(0.12)
Middle Africa	1094	46	71	81	80	88	103	126	145	164	190
	**[2.9] **(0.02)	**[1.9] **(0.01)	**[2.5] **(0.02)	**[2.8] **(0.02)	**[2.6] **(0.02)	**[2.5] **(0.02)	**[2.6] **(0.02)	**[3.1] **(0.02)	**[3.3] **(0.02)	**[3.5] **(0.02)	**[3.5] **(0.03)
Northern Africa	9248	534	529	620	735	804	1017	1005	1230	1284	1490
	**[24.7] **(0.17)	**[21.9] **(0.12)	**[18.6] **(0.12)	**[21.6] **(0.13)	**[23.4] **(0.15)	**[22.8] **(0.15)	**[25.5] **(0.19)	**[24.4] **(0.19)	**[27.8] **(0.18)	**[27.3] **(0.21)	**[27.5] **(0.22)
Southern Africa	11493	840	923	963	961	1186	1241	1205	1268	1334	1313
	**[30.7] **(0.22)	**[34.4] **(0.19)	**[32.5] **(0.21)	**[33.6] **(0.21)	**[30.6] **(0.21)	**[33.6] **(0.20)	**[31.2] **(0.23)	**[29.2] **(0.23)	**[28.6] **(0.22)	**[28.4] **(0.22)	**[29.1] **(0.23)
Western Africa	8822	360	735	718	777	821	903	1049	1011	1135	1313
	**[23.6] **(0.17)	**[14.7] **(0.08)	**[25.9] **(0.17)	**[25.0] **(0.16)	**[24.7] **(0.16)	**[23.3] **(0.16)	**[22.7] **(0.17)	**[25.4] **(0.19)	**[22.8] **(0.19)	**[24.2] **(0.17)	**[24.3] **(0.19
All	37467	2442	2839	2868	3141	3530	3983	4127	4426	4699	5412
	**[100.0] **(0.70)	**[100.0] **(0.55)	**[100.0] **(0.64)	**[100.0] **(0.62)	**[100.0] **(0.65)	**[100.0] **(0.68)	**[100.0] **(0.74)	**[100.0] **(0.74)	**[100.0] **(0.76)	**[100.0] **(0.75)	**[100.0] **(0.79)

Shown are the numbers of articles indexed by PubMed, from different Africa subregions, for the period 1996 – 2005. Values are expressed as number of articles (%) within a calendar year, and percentage of worldwide publications

**Figure 1:**
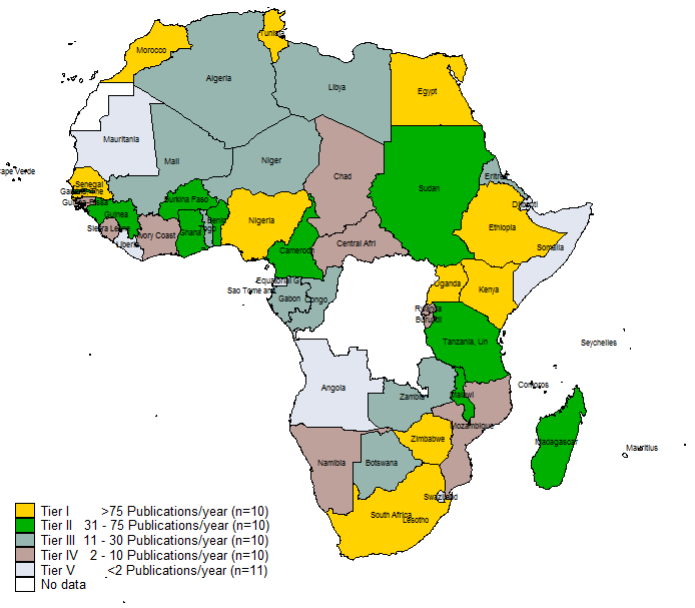
Color-coded map representing number of PubMed publications per year (1996 – 2005).

Figure 1 shows average number of PubMed publications per year broken down by quintiles. Ten countries had more 75 publications per year that put them in the highest quintile, ten belonged in the second quintile (31 – 75 publications per year), ten in third quintile (12 – 30 publications per year), ten in the fourth quintile (3–11 publications per year), and 11 countries in the lowest quintile (< 3 publications per year).

**Figure 2:**
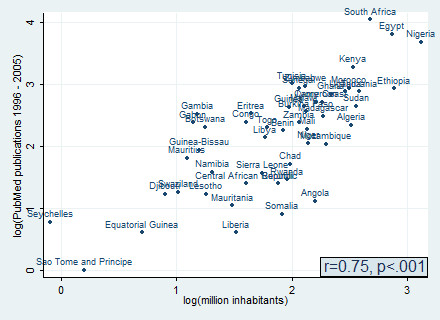
Scatter plot depicting the association between total PubMed publications for different countries in Africa and the population of each country

**Figure 3:**
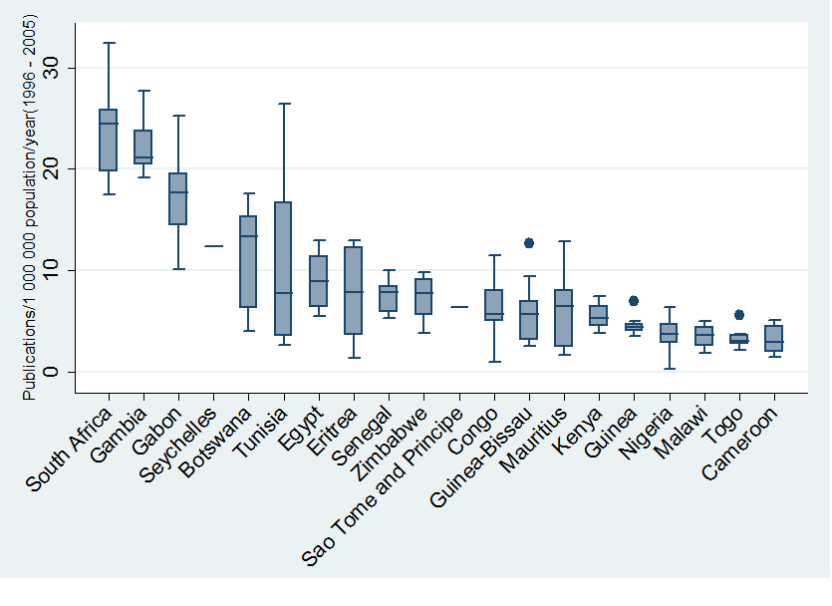
Top 20 countries and their number of PubMed publications per 1,000,000 populations per year during the period between 1996 and 2005. Horizontal lines in the box represent the 1st, 2nd (the median), and 3rd quartile; whiskers (vertical lines) represent extension of values up and down.

There was a strong positive and statistically significant correlation between the absolute numbers of published articles and countries' population (Pearson correlation r = 0.75, p < .001) (Figure 2). In absolute numbers, large countries such as the Nigeria, Egypt, and South Africa contributed more publications over the study period than smaller countries such as Equitorial Guinea, Sao Tome and Principle, and Seychelles. Figure 3 make it abundantly clear that small countries like Gambia, Gabon, and Botswana are among the countries that efficiently contributed to publications in terms of research output per million inhabitants. It is noteworthy that even with the publications related to the population South Africa still rank first.

**Figure 4:**
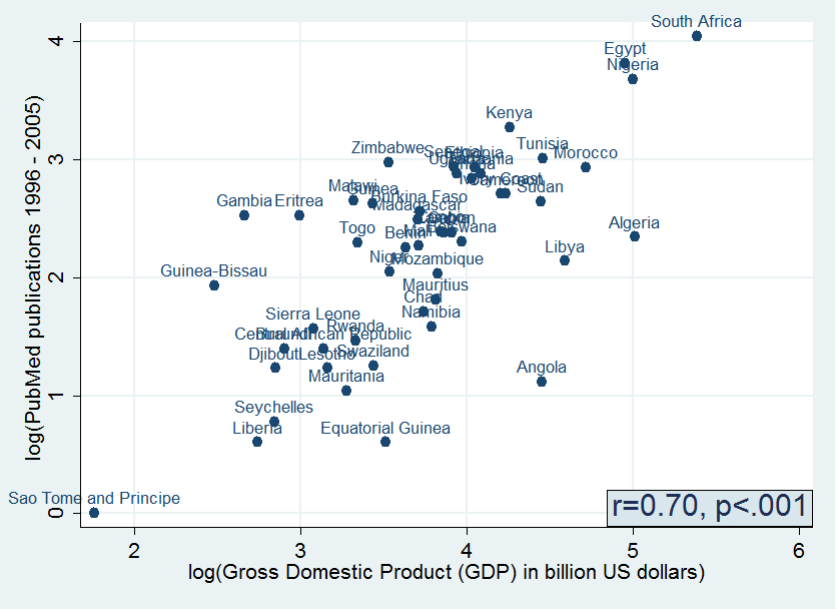
Scatter plot depicting the association between total PubMed publications for different countries in Africa and the gross domestic product (GDP) of each country in billions of 2005 US dollars.

**Figure 5:**
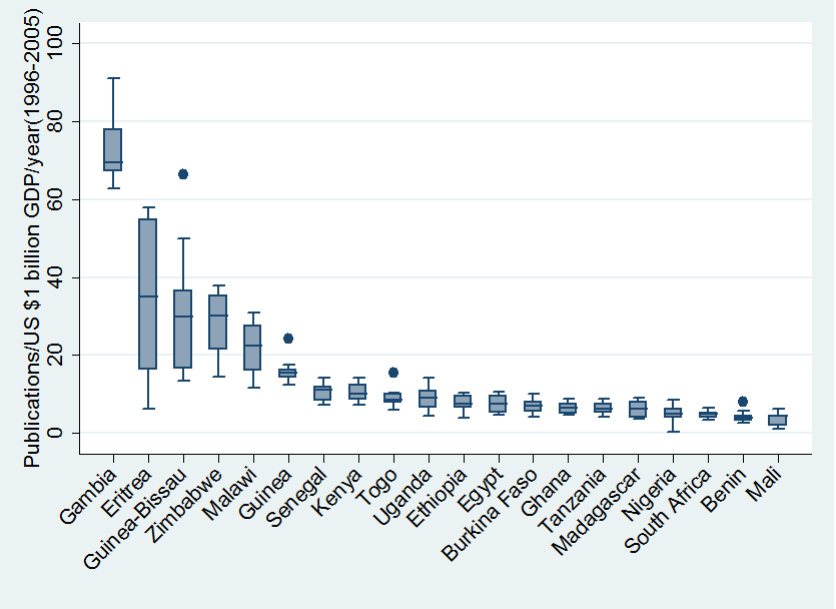
Top 20 countries and their number of PubMed publications per one billion US dollars GDP per year during the period between 1996 and 2005. Horizontal lines in the box represent the 1st, 2nd (the median), and 3rd quartile; whiskers (vertical lines) represent extension of values up and down.

In addition, there was a strong positive and statistically significant correlation between PubMed publications and countries' GDP (Pearson correlation r = 0.70, p < .001) (Figure 4). However, when total product was adjusted for GDP, we found that countries such as The Gambia, Eritrea, and Zimbabwe to be the most productive countries. Whereas countries such as South Africa and Nigeria had a lower number of PubMed publications relative to their GDP (Figure 5).

### Subregional analyses

The total production of articles in each define Africa sub-region, as well as relative contribution is displayed in Table 2. As shown in this table, Southern Africa was the most productive sub-region (30.1% of the Africa articles indexed by PubMed throughout the whole period studied). We observed continuous increase in the production of research articles from all Africa subregions during the period 1996 – 2005. As shown in Table 2, Africa's publication output trends show that its contribution to global knowledge production has been low during the period of 1996 – 2005. Southern, Northern, and Western Africa share of global biomedical science showed slight increase from a 0.19%, 0.12%, and 0.08% share in 1996 to 0.23%, 0.22%, and 0.19% in 2005. Middle Africa share has been constant over the study period, while Eastern Africa has lost 20% of it share in global biomedical publications.

There was a strong and statistically significant correlation between the absolute numbers of published articles between the different subregions during the years of the study period (1996 – 2005). The median (range) of the Pearson correlation test values for between comparisons of 45 possible couples of the specified five Africa subregions was 0.98 (0.84 – 0.99). All comparisons had statistical significance at levels < 0.05 and more specifically 36 had statistical significance at levels < 0.01.

**Figure 6:**
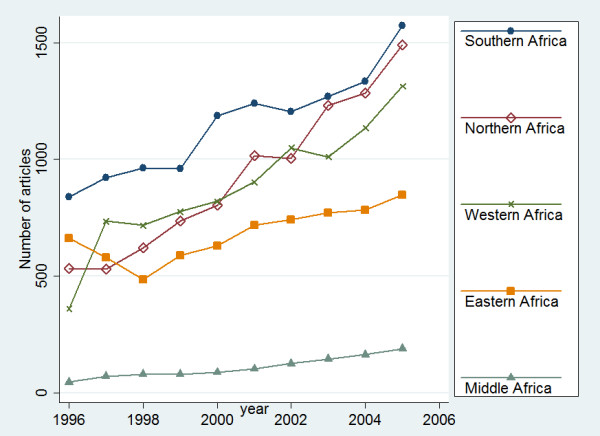
Trends in Africa subregional biomedical articles output indexed by PubMed (1996 – 2005)

Figure 6 depicts the trends of research production in the period of 1996 – 2005. Southern Africa ranks first in absolute growth followed closely by Northern Africa. African continent is very heterogeneous in terms of trend in research production (Table 3). The average annual percentage change (AAPC) of Africa subregions, show very significant differences ranging from 4.6% for Eastern Africa and 6.5% in Southern Africa, to astonishing 14.8% in Middle Africa and 12.8% in Northern Africa. Compared to model 2 (1996 – 2000), in the model 3 (2001 – 2005) AAPC for Western, Northern, and Southern decreased, whereas for AAPC for Eastern and Middle Africa increased. A non-statistically significant downward trend was observed in during 1996 – 2005 in Eastern Africa.

**Table 3:** Poisson regression analyses of average annual percent change in research production by Africa subregions.

	***Model 1 (1996 – 2005)***			***Model 2 (1996 – 2000)***			***Model 3 (2001 – 2006)***		
	**β (SE)**	**AAPC**	**P-value**	**β (SE)**	**AAPC**	**P-value**	**β (SE)**	**AAPC**	**P-value**

Eastern Africa	0.05 (0.004)	4.6	<0.001	-0.10 (0.013)	-0.9	0.474	0.04 (0.011)	3.9	0.001
Middle Africa	0.14 (0.110)	14.8	<0.001	0.13 (0.037)	13.6	0.001	0.15 (0.027)	15.8	<0.001
Northern Africa	0.12 (0.004)	12.8	<0.001	0.12 (0.013)	12.3	<0.001	0.10 (0.010)	10.7	<0.001
Southern Africa	0.06 (0.003)	6.5	<0.001	0.08 (0.010)	7.8	<0.001	0.06 (0.009)	6.2	<0.001
Western Africa	0.10 (0.004)	10.1	<0.001	0.14 (0.012)	15.3	<0.001	0.08 (0.010)	8.8	<0.001
All	0.08 (0.002)	8.7	<0.001	0.08 (0.006)	8.7	<0.001	0.08 (0.005)	7.9	<0.001

β indicates regression coefficient (trend); SE standard error of β, AAPC average annual percentage change

## Discussion

### Main findings

Our study shows that research production in Africa is highly skewed; South Africa accounts for one third of Africa's publication output, Egypt and Nigeria jointly account for another one third, result that is congruent with the findings of previous studies [21,31]. In this study, we found like others [1,2,21,32-34], that highly populous countries have corresponding high levels of PubMed publications; and smaller countries can very productive. Taking into consideration of their GDP, we found that the better the economic ranking of a country the higher the quantity of its biomedical publications. The correlation between research productivity and GDP has received some attention in the past [2,33,35-40]. We recognise that other factors such number of journals, high number of research institutions or universities, and research specialization may also have contributed to the observed high performance of the top 3 countries (South Africa, Egypt, and Nigeria). Tijssen [21] argued that the international research production of African science is partly dependent upon their research specialization profile. Some fields of science, like the medical sciences, are internationally oriented and tend to attract more international funds, partnerships, and opportunities to publish in international scientific literature.

Another interesting finding in our study was the reduction in relative contribution of South Africa over the period studied. This finding was also observed in the past by other investigators [21]. Contrary to previous research [41], Nigeria exhibited the most significant relative growth in number of PubMed publications over the study period; this finding is intriguing and would need benefit from further exploration. This and a previous study [21] found that Tunisia accounted for the modest average annual percentage change. Language barriers did not seem to be a significant impediment with substantial PubMed contributions in English publications from authors from Arab speaking nations (such as Tunisia and Morocco) and Francophone Africa (such as Senegal and Cameroon). It is possible that Anglophone collaborators are contributing to shaping these publications.

On subregional analyses, Southern Africa is the most productive sub-region in terms of absolute number of articles indexed by PubMed. During this 10-year period, Southern and Northern Africa had the best trends of total product of research production. We observed continuous increase in the production of research articles from all Africa subregions during the period 1996 – 2005. Tijssens [21] found that Africa research output has risen by 38% up to 46, 000 articles in 2001 – 2004. One explanation for could be that the electronic online submission systems during last years made easier for African authors to submit their studies [39].

Despite the upward trend, we found like other that contribution of Africa to global research production was limited [21]. In addition to less research being done in Africa, other likely reasons for this limited number of publications are low research and development expenditure [42], poor research methods, and problems of presentation such as writing style and language competency [43]. Serious under-representation of editorial and advisory board members from countries with a low human development index in general medicine [44], tropical medicine [5], medical education [45], psychiatry [43,46], and anaesthesia/critical care [47] journals has been documented recently.

### Study limitations

Our study had several limitations that should be kept in mind when interpreting the results. Most of the limitations are related to the database used to retrieve articles as this consist largely of English-language journals therefore possibly contributing to selection bias due to language barriers. In addition, PubMed do not represent all scientific and biomedical journals published. Many articles of biomedical importance appear in journals other than those we included in our searched categories. Gaillard [14] argues that some 65% of African research papers are published in local journals that are not listed in the international citation databases. As noted by Tijssen [21], the current information sources and quantities indicators inevitably offer incomplete pictures of the strength and weaknesses of Africa biomedical publications. Regrettably, many research publications by African researchers, especially those focused on domestic or regional African issues and problems, are not assessable through modern ICT facilities (electronic databases, Internet facilities). Therefore are often neglected in assessment of Africa science.

Other limitations include the incorrect citation of origin for the authors, and definition of research production. By using the author addresses listed in the bylines of research articles, one can only identify countries and organizations where the authors were employed when the research was done or where the article was written, or both. Institutionally co-authored research articles co-publications are useful and tangible proxies of research involving African scientists and scholars. We have focused in this report on the number of publications as a measure of research output. Obviously, there are other variables that also describe research productivity such as impact factors, citation index, conference presentations, grants, etc, that need to be studied in any future phases of this study.

Finally, we should emphasize that the division of the Africa into different subregions could be done in various ways. Our classification was based on several criteria, but alternative approaches would also be appropriate. For example, Africa could be grouped into Northern Africa and Sub-Saharan. In addition, we could have used the official language to group Africa countries.

### Policy implications

We believe like others [26,48-59] that scientific knowledge and understanding of diseases could significantly be benefited should the developing countries be provided with additional resources to improve their research productivity. Results of this study have several policies implications:

1. Research funding agencies should bear in mind that unpublished research has limited or perhaps even no value [26]. Re-thinking polices and procedures to ensure dissemination of research to health professionals should include a broader definition of funding for research. Either consideration should be given to providing resources or allocating funds in the budget of grants to promote the expertise of key stakeholders in the research cascade including authors, reviewers, and editors in the region.

2. Developing computerized knowledge management systems to more accurately track research output including the grey literature [26,51,52].

3. Research institutions should use their health diaspora more strategically. Exploiting this community as a source of peer reviewers could provide journals with a previously untapped source of expertise. This would have the net effect of strengthening local journals and by promoting "brain circulation" would keep researchers abroad scientifically connected to their homeland [26].

## Conclusion

In summary, we examined one decade of publication trends by first authors in Africa from 1996 to 2005 in PubMed index journals. The results of our study showed that South Africa, Egypt, and Nigeria were the most productive countries in terms of absolute number of publications indexed by PubMed. As expected, the most populous and rich countries have corresponding higher production. However, South Africa and Gambia had the best performance based on number of research articles per million inhabitants; while The Gambia and Eritrea had the best performance based on number of research articles per GDP. We observed continuous increase and re-assuring trends in the production of research articles from all Africa subregions during the period 1996 – 2005. However, contribution of Africa to global research production was rather limited. For African countries to achieve prolonged significant growth there is a need to embark on economic catch-up trajectories, sustained capacity-building, investments, and upgrading of their science bases.

## Competing interests

The author(s) declare that they have no competing interest.

## Authors' contributions

OAU was involved in the conceptualization, research design, analysis and write-up of the draft of the manuscript. MU assisted in interpretation of findings and manuscript preparation.
